# PAM (PIK3/AKT/mTOR) signaling in glia: potential contributions to brain tumors in aging

**DOI:** 10.18632/aging.202459

**Published:** 2021-01-05

**Authors:** Michael R. Duggan, Michael Weaver, Kamel Khalili

**Affiliations:** 1 Department of Neuroscience Lewis Katz School of Medicine at Temple University Philadelphia, PA 19140, USA; 2 Department of Neurosurgery Temple University Hospital Philadelphia, PA 19140, USA

**Keywords:** glioma, aging, bioenergetics

## Abstract

Despite a growing proportion of aged individuals at risk for developing cancer in the brain, the prognosis for these conditions remains abnormally poor due to limited knowledge of underlying mechanisms and minimal treatment options. While cancer metabolism in other organs is commonly associated with upregulated glycolysis (i.e. Warburg effect) and hyperactivation of PIK3/AKT/mTOR (PAM) pathways, the unique bioenergetic demands of the central nervous system may interact with these oncogenic processes to promote tumor progression in aging. Specifically, constitutive glycolysis and PIK3/AKT/mTOR signaling in glia may be dysregulated by age-dependent alterations in neurometabolic demands, ultimately contributing to pathological processes otherwise associated with PIK3/AKT/mTOR induction (e.g. cell cycle entry, impaired autophagy, dysregulated inflammation). Although several limitations to this theoretical model exist, the consideration of aberrant PIK3/AKT/mTOR signaling in glia during aging elucidates several therapeutic opportunities for brain tumors, including non-pharmacological interventions.

## INTRODUCTION

Cancer in the brain is significantly less common than in other organs, but disproportionately contributes to higher rates of mortality [1, 2]. Although prognostic factors have been identified (e.g. MGMT promoter methylation), long-term treatments remain ineffective, in part due to diverse challenges in research and development as well as an incomplete understanding of brain tumor biology [3–5]. Treatment options for central nervous system (CNS) tumors are limited (e.g. chemotherapy, radiotherapy (RT), surgical resection etc.) and overall mortality rates exceed 60% within 5 years of diagnosis [6].

In the United States, the prevalence of brain tumors is highest in the 50-59 age group, followed by the 60-69 and 70-79 cohorts, respectively [7]. While the frequency of older individuals diagnosed with these insidious neuropathologies continues to increase, advancing age itself is associated with poorer prognosis [8–12]. For instance, elderly patients maintain elevated mortality rates compared to younger individuals and benefit from shorter courses of RT because of increased susceptibility to side effects of full-dose RT [12, 13]. Given their vulnerability, as well as the contemporary increase in aging demographics, determining the underlying biological mechanisms of tumor pathology in the aging brain and improving treatment options has never been more urgent [14]. Since metabolic syndrome (e.g. hyperglycemia) is an independent risk factor for worse prognosis in patients with certain CNS tumors, bioenergetic processes in particular are emerging as important variables in tumor neuropathology [15].

Among primary cell types implicated in tumor pathogenesis, minimal contributions are observed from non-glia; less than 10% of all tumors manifest as lymphomas, meningiomas, embryonal tumors, or choroid plexus carcinomas, among others [6]. Although neuron-specific oncogenesis has been documented (e.g. neurocytoma, ganglioneurocytoma), its prevalence is extremely rare (~1%) and is routinely characterized by mixed neuronal-glial presentation [16]. Additionally, while microglia are sufficient to augment tumor progression in the CNS, microglia-specific cancers appear to be absent [17, 18]. Conversely, more than 75% of cancers in the CNS are of glial origin [6]. These tumors can be classified and graded in severity utilizing an array of molecular markers and assessment of specific genetic mutations (e.g. isocitrate dehydrogenase; IDH) [19]. Within glioma subtypes, glioblastomas account for more than half of all diagnoses and are the most lethal, with a 5-year survival rate of less than 6% [20]. As tumors of glial origin account for the vast majority of all CNS cancers (i.e. pilocytic astrocytoma, oligodendroglioma, diffuse astrocytoma, anaplastic astrocytoma, glioblastoma), age-dependent variation in glial functioning may particularly contribute to tumor pathology in the brain [6].

The unique role of glia in modulating CNS bioenergetics is often underappreciated and is reminiscent of the metabolic characteristics found in cancer cells of other organs. Here, glia can preferentially process glucose to produce lactate, which is then exported to serve as a primary energy source for energy-demanding neuronal populations [21, 22]. Such predominant production of lactate and its subsequent extrusion to neurons, rather than glucose utilization in mitochondrial oxidative phosphorylation, is indeed similar to the metabolic characteristics commonly observed in cancer cells in other organs (i.e. Warburg effect; aerobic glycolysis) [23, 24]. Within the CNS, astrocytes in particular have long been associated with metabolic homeostasis and are the most common cell type associated with tumor pathology [6, 25, 26]. This suggests the unique metabolic processes and related pathways in these glia may contribute to glioma pathology in the aging CNS as well as poorer outcomes in aged patients.

Along with a continual production of lactate, cancer cells often maintain hyperactivation of the PAM (PI3K/AKT/mTOR) pathway [27, 28]. This evolutionary conserved metabolic pathway can be governed by upstream insulin signaling and may be one of the most diverse in mammals; it affects a range of cellular molecules and functions, from nutrient transporters and metabolic enzymes to gene expression and autophagy, respectively [29, 30]. Interestingly, variation in bioenergetic demands, insulin signaling (e.g. insulin resistance) and downstream PAM signaling (e.g. mTOR dysregulation) are characteristic of the aged brain and are associated with age-related neuropathogenesis [31–34]. Given that glia are responsible for maintaining energy homeostasis of the CNS by utilizing similar metabolic pathways of cancer cells (i.e. aerobic glycolysis), chronic alterations in bioenergetic demands during aging may aberrantly regulate PAM-dependent mechanisms in glia (e.g. cell cycle progression, autophagy, neuroinflammation); in turn, this could contribute to tumor pathology in the CNS and facilitate poorer prognosis in the elderly.

## Cancer bioenergetics and PAM signaling

The metabolism of cancer cells is distinct and the implications of these bioenergetic characteristics are increasingly relevant in assessing the etiology of oncogenesis as well as tumor development [35, 36]. For instance, dietary high-fructose corn syrup was recently shown to directly contribute to tumor formation in murine intestines [37]. Similar to glia in the brain (see below), glucose in cancer cells is predominantly converted to lactate via glycolysis, rather than being fully oxidized via respiration in mitochondria. The increased glucose flux and conversion to lactate despite the presence of adequate oxygen levels (i.e. aerobic glycolysis) as well as functional mitochondria has been termed the “Warburg Effect” [24, 38, 39]. While this produces a higher rate yet lower yield of ATP production, it is also thought to generate substrates that facilitate anabolic processes during cell proliferation (e.g. lipids, proteins, nucleotides), promote intercellular oncogenic signaling (e.g. reactive oxygen species; ROS) and disrupt functioning in surrounding non-oncogenic cells [39–42]. Coupled with altered vasculature as well as a continuous supply of glucose and glycolysis-promoting factors (e.g. insulin), such distinct metabolism of cancer cells promotes their fitness at the expense of their non-oncogenic neighbors [43, 44].

In addition to metabolic reprogramming, cancer cells routinely display hyperactivation of Phosphoinositide 3-kinase (PI3K)-dependent pathways, a signaling cascade that controls multiple steps in cellular bioenergetics and can promote aerobic glycolysis [27, 45]. PI3K, and its downstream effectors AKT/mTOR (PAM), constitute an evolutionarily conserved metabolic pathway that can be modulated by an array of factors, including insulin signaling [46]. In brief, insulin or other growth factors (e.g. insulin-like growth factors; IGF) bind to requisite receptors on the cell surface, causing insulin-receptor substrate phosphorylation, downstream PI3K/AKT/mTOR activation and ultimately increased glucose uptake [28, 47]. Consequently, hyperinsulinemia induced PAM activation can promote tumor progression and is associated with increased mortality rates in non-CNS cancers [48–52]. Conversely, the mitigation of insulin levels in non-CNS cancer cells can inhibit oncogenesis, while insulin-induced proliferation in these cells can be reversed by inhibiting PAM activation [53, 54].

Given its diverse roles in maintaining cellular bioenergetics, aberrant PAM signaling in cancer is associated with variation across several interdependent homeostatic mechanisms, including cell cycle progression, autophagy and inflammation. For instance, activation of PI3K and subsequent phosphorylation of AKT is necessary for mitigating the inhibitory regulation of cyclin-dependent kinases in cellular proliferation, thus promoting cell cycle entry in cancer cells [55, 56]. As a potent autophagy inhibitor, mTOR1 is also a notable downstream target of PI3K activation in cancer [57, 58]. Specifically, mTOR1 can suppress a variety of rate limiting steps in this protein degradation system, including the inhibition of autophagosome dynamics (e.g. nucleation, elongation, termination) and the dysregulation of transcription factors responsible for the expression of autophagy-dependent genes (e.g. TFEB) [59, 60]. Additionally, PAM activation can dysregulate host immune mechanisms and promote tumor progression, while the pharmacological inhibition of PI3K can mitigate these processes [61–63]. For instance, PI3K activation inhibits anti-cancer capacities in T-cells to promote tumorigenesis, and PI3K inhibition can mitigate the recruitment of myeloid cells to the tumor microenvironment that otherwise promote tumor growth [64–66]. Thus, the metabolic demands of oncogenic cells and associated hyperactivation of PAM signaling may potentiate pathogenesis through these altered homeostatic mechanisms.

## Glia bioenergetics and PAM signaling

The energy demands of the CNS necessitate efficient regulation and are uniquely dependent on glia. Indeed, the brain requires more energy compared to other organs (i.e. 2% of body mass but 20% of total body energy), the demands between cells are disproportionate (i.e. 80-90% of all energy in the brain is used by neurons for action potential generation, maintaining resting membrane potential etc.) and its lack of reserves (e.g. limited adipose tissue) must be compensated by continuous access to essential macronutrients [67–70]. Although neurons themselves have been shown to process glucose for energy, efficient bioenergetics in the CNS involve glucose conversion to lactate by glia, which is then extruded to serve as a principal energy source for neuronal populations [21, 22].

In a process termed the astrocyte-to-neuron lactate shuttle (ANLS) hypothesis, astrocyte pericapillary end-feet initially take up glucose from the resource-rich vasculature in conjunction with pericytes, convert it to lactate via lactate dehydrogenase (LDH) and transport the lactate via monocarboxylate transporters (MCT) to neurons, where it serves as an energy substrate for ATP synthesis in neuronal mitochondria [23, 71]. Such metabolic processes are linked to activity dependent neurotransmission, where evoked release of vesicular glutamate at distal axonal terminals is taken up by astrocytes, converted to glutamine and transported back to neurons, which are otherwise incapable of *de novo* synthesis of such neurotransmitter precursors [72–74]. Along with fluctuations in metabolic demands, neuronal activity may directly contribute to tumor pathogenesis via glutamate signaling and other small molecules produced by neuronal firing (e.g. neuroligin-3) [75, 76]. As tumor growth induced by neuronal activity coincides with cancer cell colonization at synaptic junctions (i.e. where astrocytes would otherwise be located given the ANLS), further investigations are encouraged to determine how differing functioning of glia during aging may alter this interaction with neurons to facilitate tumor development [77].

Although direct evidence for some of these mechanisms is lacking, and other processes undoubtedly contribute to energy homeostasis in the CNS (e.g. neuronal oxidative phosphorylation and lactate export, pentose phosphate shunt, glycogen turnover, acetate use etc.), accumulating evidence implicates the importance of glycolysis in astrocytes for maintaining CNS bioenergetics [21, 78–80]. More recently, similar lactate-dependent bioenergetic processes have been extended to oligodendrocytes, the second most common cell type associated with CNS cancers [81–83]. It should be noted that precursors of both astrocytes -and oligodendrocytes may serve as progenitors for tumorigenesis [84, 85]. Given that astrocytic support for oligodendrocytes via extracellular vesicles is compromised in aging, future experiments should examine if age-related metabolic variations influence the interplay between astrocytes and oligodendrocytes to promote pathological processes in CNS cancers [86].

While the metabolic demands of glia are unique in the CNS and emulate cancer metabolism (e.g. glycolysis instead of respiration), variation in PAM signaling can also accompany these metabolic variations in the absence of overt pathogenesis. In astrocytes, enhanced lactate production triggered by oxidative stress is dependent on PAM activation [87]. Similarly, insulin stimulation of astrocytes results in a dose-dependent activation of downstream PI3K-dependent effectors, including AKT and GSK3 [88]. Although evidence of differing glycolysis associated with PAM variation in non-oncogenic oligodendrocytes is lacking, increased metabolic demands in these cells (e.g. myelination) are indeed associated with increased PI3K-dependent signaling [89, 90]. Moreover, metabolic demands of astrocytes and ensuing activation of PAM signaling can precipitate similar signaling cascades in their oligodendrocyte counterparts [91].

Elevated glucose uptake, increased glycolysis and altered metabolic pathways are well characterized features of glioma biology, similar to other cancer types [92, 93]. Such elevated glycolysis is most commonly associated with genetic dysregulation of PAM pathways, which are evident in over 85% of glioblastoma cases and can predict prognosis in patients [94, 95]. While aberrant PAM activation accompanies oncogenesis across various glioma subtypes, the induction of PAM signaling *in vitro* is sufficient to promote cellular proliferation in human glioblastoma as well as oligodendroglioma cells [96–100]. Experiments utilizing similar cell culture systems suggest aerobic glycolysis and sufficient lactate production in brain tumors is inherently dependent on PAM pathway induction, while pharmacological inhibition of this signaling pathway in the brains of mice can mitigate the growth of orthotopic tumors [101, 102]. Evidence also suggests the involvement of PAM is conserved across tumors of varying glial origin, provided that *in vivo* inducible expression of PI3K is capable of precipitating oligodendroglioma oncogenesis, while deletion of PI3K-inhibitory factor (PTEN) can facilitate substantially more aggressive tumor progression [103].

Similar to other cancers, dysregulated PAM signaling in glioma cells is associated with variation across several important homeostatic mechanisms, such as cell cycle progression, autophagy and inflammation. In glioblastoma cell lines (i.e. U87MG and U251MG), and *ex vivo* glioma brain slices, activation of PAM signaling is necessary for cell cycle progression and tumor expansion, potentially due to PI3K/AKT-suppression of cyclin D1 [98, 104]. Additionally, glioma cells require PAM-induced suppression of autophagy to maintain viability, while suppressed autophagy and tumor infiltration in orthotopic glioma models is dependent on PAM activation [105, 106]. Glioma similarly activates PAM pathways to modulate cytokine signaling and recruit proinflammatory microglia to the microenvironment, which promote tumor growth [107–111]. Given the unique metabolic demands of glia (i.e. aerobic glycolysis) and the dysregulation of PAM signaling observed in glioma cells, variation in CNS bioenergetics during aging and associated PAM signaling in glia may contribute to pathological processes in CNS cancers.

## Potentiation of glial PAM in aging: potential mechanisms in brain tumors

Due to their role in maintaining energy homeostasis in the brain, alterations in CNS bioenergetics that accompany aging may modulate the metabolic demands of glia and dysregulate their PAM signaling, thus contributing to tumor pathology in elderly patients. Dysregulation of insulin and glucose processing in the CNS is characteristic of aging as well as age-related neurodegenerative diseases (e.g. Alzheimer’s Disease, AD; Parkinson’s Disease, PD) [112–115]. Such deficiencies in energy dynamics during aging are due to several factors, including impairments in mitochondrial oxidative phosphorylation, altered expression of glucose transporters, dysregulated redox homeostasis, and adaptations to insulin signaling itself [116–119]. Furthermore, a limited nutrient supply and hypoperfusion may exacerbate the disproportional strains on aerobic glycolysis in aging [120, 121]. While deficits in energy utilization are characteristic of the aging CNS, astrocytes can compensate for metabolic deficiencies in neurons (e.g. increase glucose uptake, upregulate glycolysis) [122–124]. Interestingly, variation in these bioenergetic demands can trigger a direct upregulation in glial glycolysis, sometimes referred to as the “Inverse Warburg Effect” [125]. Indeed, astrocytes can better cope with hyperglycemic fluctuations compared to neurons, and they can directly alter their metabolism as well as PAM signaling in response to insulin stimulation [88, 123, 126].

**Figure 1:**
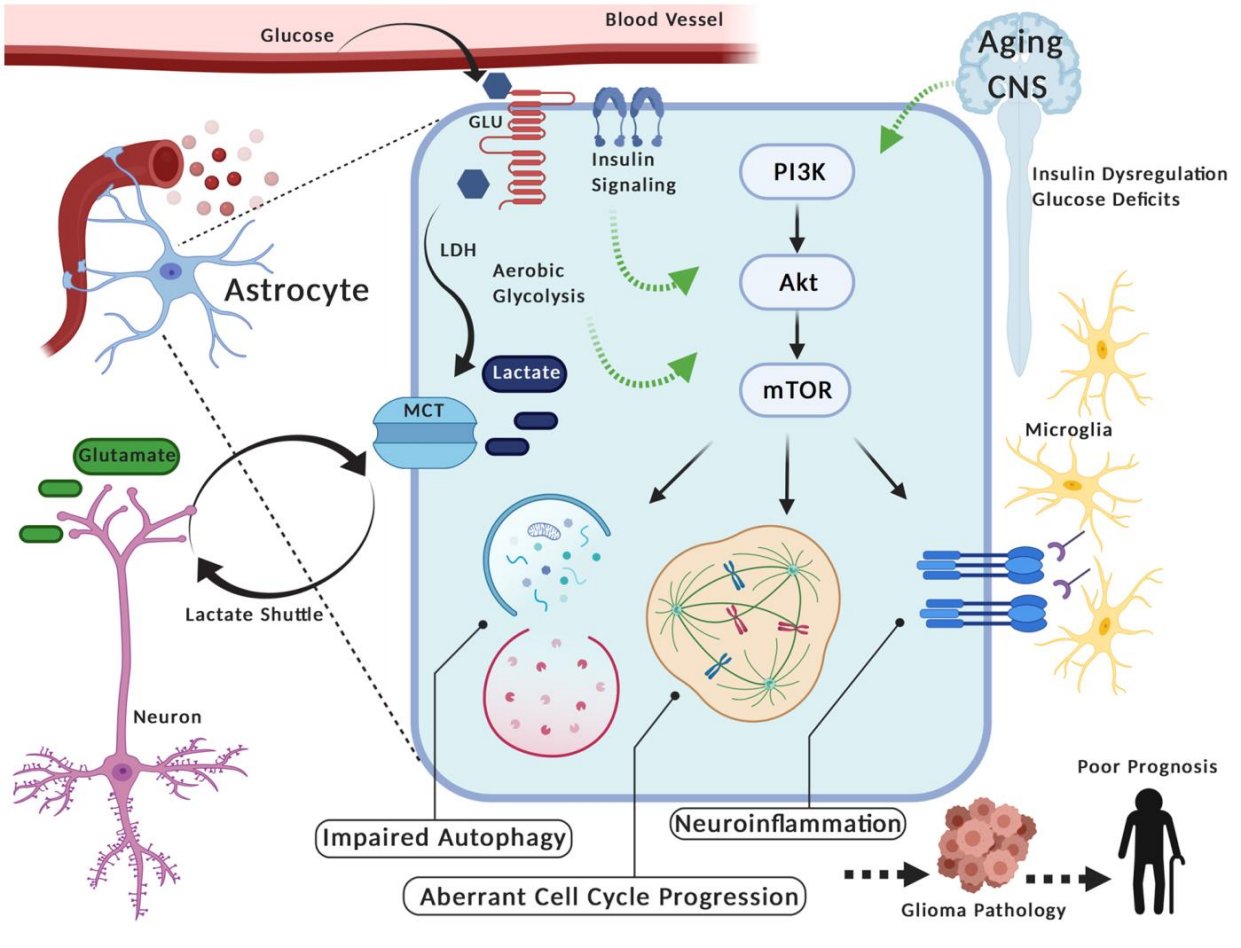
**PAM (PIK3/AKT/mTOR) Signaling in glia during aging may contribute to glioma pathology.** Astrocytes take up glucose, convert it to lactate (i.e. aerobic glycolysis) via lactate dehydrogenase (LDH) and transport it via monocarboxylate transporters (MCT) to neurons (i.e. Lactate Shuttle) where it serves as an energy substrate. These glia can alter their metabolism and also re-uptake glutamate in response to neuronal firing activity. Interestingly, the aerobic glycolysis in astrocytes is reminiscent of energy production in cancer cells, including gliomas. Furthermore, variation in metabolic activity and/or insulin stimulation in astrocytes corresponds with activation of PAM (PIK3/AKT/mTOR), a pathway linked to oncogenic processes in tumor cells (e.g. aberrant cell cycle progression, impaired autophagy, neuroinflammation). Thus, metabolic fluctuations in the central nervous system (CNS) during aging (e.g. insulin dysregulation, deficits in glucose utilization) may aberrantly potentiate PAM signaling in these glia, given their prominent role in metabolic homeostasis. In turn, this could contribute to pathogenic processes of gliomas and facilitate poor prognosis in elderly individuals. Created with Biorender.com.

Given the role of glia, specifically astrocytes, in maintaining metabolic homeostasis in the CNS, variation in such metabolic demands during aging may aberrantly regulate PAM signaling in these cells to promote tumor pathology. Indeed, elevations of insulin can trigger PAM signaling in astrocytes, while the induction of aerobic glycolysis in astrocytes is itself dependent on PAM activation [87, 88]. However, astrocytic adaptations to these conditions may be maladaptive. For instance, disrupted insulin signaling in astrocytes following metabolic fluctuations may inhibit the transport of insulin/glucose into the brain [112, 127]. In addition, cell cycle progression in astrocytes can be modulated by hyperglycemic conditions, while proliferation and survival of CNS cancer cells is inherently coupled to insulin-dependent PAM signaling [100, 128–130]. Furthermore, accumulating evidence indicates certain subpopulations of astrocytes with distinct metabolic characteristics are more prone to oncogenesis, while such glial heterogeneity significantly contributes to tumor development [131, 132]. Conversely, the reduction in glycolysis at the expense of increased oxidative phosphorylation (i.e. “Anti-Warburg Effect”) is sufficient to trigger glioma differentiation into non-oncogenic astrocytes [133]. Moreover, whereas the induction of PAM signaling is observed in glial pathogenesis and tumor development, its mitigation is associated with extended longevity and CNS homeostasis in aging [31, 134, 135]. Therefore, metabolic inefficiencies in the brain during aging may converge on glial bioenergetic demands to dysregulate PAM-dependent mechanisms, promote tumor pathology, and contribute to poor prognosis in elderly patients (Figure 1).

Similar to observations in gliomas, dysregulation of PAM-dependent mechanisms is observed in age-related neurodegenerative conditions, suggesting their contributions to CNS disease progression in aging. Indeed, PAM induction of aberrant cell cycle progression, impaired autophagy and neuroinflammation is implicated in AD pathogenesis [136–138]. Additionally, data suggest that patients displaying AD symptomology maintain poorer cancer-related outcomes, including diagnosis with more advanced stages of cancer, decreased life expectancies and increased mortality rates [139–143]. Such activation of PAM-dependent mechanisms and downstream oncogenesis is seemingly contradicted by population-based cohort studies, which indicate AD patients maintain reduced risk of cancer by up to ~50% and cancer patients maintain reduced risk of AD by up to ~35% [144, 145]. However, it is important to note this correlation may be confounded by sampling errors in data collection (i.e. demented patients are less likely to be screened for cancer and cancer patients may not live long enough to develop dementia) [146, 147]. Therefore, PAM signaling may nonetheless represent an important node of CNS homeostasis in aging, whereby its dysregulation can facilitate impairments in crucial mechanisms that promote disease progression in the brain.

In addition to PAM, age-related variation in several other glial mechanisms may influence glioma pathology and contribute to poor prognosis among elderly patients. For instance, aging astrocytes can develop distinct expression profiles, characterized by upregulation of multiple immune pathways (e.g. complement system, antigen presentation), increased immune cell attractants (e.g. CXCL5, CXCL10) and elevations in proinflammatory cytokines [148–150]. Given that variation in neuroinflammatory microenvironments can augment tumor progression, the inflammatory profiles of aged astrocytes may facilitate glioma pathology and influence cancer-related outcomes in aged individuals [151, 152]. Although balanced ROS levels are necessary for optimal functioning, astrocytes from the aging brain also display increased levels of oxidative stress compared to their youthful counterparts, which may exacerbate the elevated levels of reactive oxygen species otherwise induced by metabolically demanding glioma cells; this suggests the mitigation of oxidative stress in aging glia may serve as a novel target for treatment of glioma in the elderly [153, 154].

## CONCLUSIONS

The present mechanistic model suggests variation in metabolic demands and aberrant PAM signaling in glia during aging, as well as its downstream consequences (i.e. cell cycle progression, impaired autophagy and neuroinflammation), are contributing factors to glioma pathology in elderly patients. Current postulations argue that tumor development may be attributed to aberrantly self-renewing, stem-like cells found within gliomas, or hypermutations in oncogenic pathways [155–157]. The latter theory is supported by observations of deficient DNA repair mechanisms in some malignancies, as well as the targeting of DNA repair mechanisms in commonly used glioma chemotherapy (e.g. TMZ) [158, 159]. Interestingly, recent evidence suggests the characteristic genetic diversity of varying glioma pathologies is likely due to culminated transcriptomic and epigenomic modifications gained from differing growth environments [160, 161]. Thus, by suggesting the unique growth environment and bioenergetic demands of glia in the aged CNS potentiate tumor pathology via PAM signaling, our model complements existing theories of glioma etiology.

The emphasis on host cell metabolic demands in aging as an important factor in glioma development supports prior data which suggests these demands can interact with viral oncoproteins to promote tumorigenesis [162]. Although neurotropic viruses encode protein antagonists of innate immune mechanisms to evade detection of viral RNA or DNA, these can inadvertently inhibit tumor suppressors and facilitate tumor progression [163–165]. For instance, p53 can be targeted by protein products in multiple viruses, including polyomaviruses (e.g. JCV), retroviruses (e.g. HIV, SIV) and herpesviruses (e.g. CMV) [166–169]. As one of the most commonly detected viruses in CNS tumors, the JCV encoded T-antigen has been shown to inactivate p53’s tumor suppressing functions and promote cell cycle progression, while these capacities of T-antigen can be independently regulated by glucose availability or insulin signaling cascades [170–173]. Furthermore, this inactivation of tumor suppressors by neurotropic viruses, in addition to their capacity to induce aerobic glycolysis and dysregulate PAM signaling, may interact with metabolic demands in aging glia to promote tumor development in the brain [174, 175].

Our model maintains several limitations. First, the role of glia themselves in regulating CNS bioenergetics remains actively debated, with some arguing that fluctuations in neuronal metabolism (e.g. glucose uptake, oxidative phosphorylation, lactate export etc.), rather than variations in astrocytic glycolysis, are predominantly responsible for energy homeostasis in the brain [80]. Additionally, while the current review was intended to highlight the unique position of glia as a potential intermediary between PAM signaling and age-related metabolic demands, this parsimonious framework does not incorporate the involvement or crosstalk with other potential pathways in glioma, particularly the MAPK/ERK cascade [176, 177]. Third, while this model takes into account the molecular signatures of glioma in patients, as well as preclinical gain/loss of function experiments, the limited empirical support for the proposed framework is underscored by the broader discordance between neurobiological and cancer research, which hinders the assessment of causal molecular mechanisms [3].

Although limitations persist, the modulation of PAM signaling in glia during aging may elucidate several therapeutic opportunities for brain tumor treatment, including the beneficial role of non-pharmacological interventions. In particular, the inhibition of PI3K’s downstream effectors, AKT and mTOR, may prove to reliably mitigate proliferation, motility and viability in glioma cells [178–180]. Additionally, PI3K inhibitors are emerging as potent antagonistic agents in various types of cancer, including gliomas [102, 181, 182]. While cancer cells have been shown to overcome this inhibition, evidence from preclinical models suggest the combination of dietary manipulations as well as PI3K inhibitors can augment the inhibition of PAM signaling and increase therapeutic efficacy [28]. Compared to mice treated with PI3K inhibitors alone, mice maintained on a ketogenic diet during treatment display significantly decreased mortality rates following intracranial implantation of aggressive human gliomas [183]. Indeed, effective regulation of insulin levels via a balanced diet or aerobic exercise is a recognized strategy to prevent age-related risks for cancer as well as neurodegenerative diseases [184–186]. Although further validation of these targets is required, the modulation of PAM signaling to mitigate glioma progression in the aging CNS may prove efficacious and merits consideration.
